# Isolation and identification of bacteria from fresh guava (*Psidium guajava*) sold at local markets in Mymensingh and their antibiogram profile

**DOI:** 10.14202/vetworld.2018.1145-1149

**Published:** 2018-08-23

**Authors:** Md. Atiqur Rahman Sarker, Md. Mazedul Haque, Rafia Afroze Rifa, Fateha Akther Ema, Md. Ariful Islam, Mst. Minara Khatun

**Affiliations:** Department of Microbiology and Hygiene, Bangladesh Agricultural University, Mymensingh-2202, Bangladesh

**Keywords:** antibiogram profile, bacteria, guava, identification, isolation, Mymensingh

## Abstract

**Aim:**

The study was conducted for the isolation, identification, and antibiogram of bacteria obtained from fresh guava (*Psidium guajava*).

**Materials and Methods:**

A total of 25 fresh guavas were collected from five markets located in Mymensingh city. Guava samples were cultured onto various selective media such as eosin methylene blue, xylose lysine deoxycholate, thiosulfate-citrate-bile salts-sucrose, blood agar, and mannitol salt agar for the isolation of bacteria. Biochemical tests (dextrose, maltose, lactose, sucrose, mannitol, methyl red, Voges–Proskauer, and indole) were performed to identify the bacteria.

**Results:**

Total viable counts of guava were ranged between log 6.56 colony-forming unit (cfu)/ml and 6.62 cfu/ml. A total of 106 bacterial isolates belonged to five genera (*Escherichia coli, Salmonella* spp., *Vibrio* spp., *Bacillus* spp., and *Staphylococcus* spp.) were identified. *Salmonella* spp. (23.6%) was the most prevalent, followed by *E. coli* (22.64%), *Bacillus* spp. (19.81%), *Staphylococcus* spp. (17.92%), and *Vibrio* spp. (16.03%). The results of antibiotic sensitivity test showed that *Salmonella* spp., *Bacillus* spp., and *E. coli* were sensitive to chloramphenicol, ciprofloxacin, and gentamicin and resistant to ampicillin and cephalexin. *Vibrio* spp. was sensitive to chloramphenicol and gentamicin, intermediately sensitive to ciprofloxacin and ampicillin and resistant to cephalexin.

**Conclusion:**

The results of this study indicate that fresh guava contains multidrug-resistant bacteria which might pose a public health risk.

## Introduction

Among indigenous fruits, guava is one of the popular fruits in Bangladesh. Guava is often called the “apple of the tropics” belonging to the family Myrtaceae and the scientific name is *Psidium guajava*. Guava stands fifth in production among the most important fruit crops of Bangladesh and is grown all over the country. According to “Yearbook of Agricultural Statistics-2015,” annual guava production in Bangladesh during 2014-2015 from inside and outside garden is 2,06,425 metric tons [1]. Guava has excellent medicinal, pharmacological, digestive, and nutritive values with pleasant flavor, high palatability, and availability in abundance at a moderate price [2].

Guava is a fresh fruit which is consumed raw without processing, thus posing a potential food safety problem by any pathogenic microorganism. Improper handling can damage fresh produce, rendering the product susceptible to the growth or survival of spoilage and pathogenic microorganisms. The guavas provide a great problem in storage and transportation because of their perishable nature [3]. In addition to inherent activities, fruits are exposed to microbial contamination through contact with soil, dust, and water. As a result, guava fruits harbor a diverse range of microorganisms [4]. Hence, in this viewpoint, assessing the microbial spectrums of guava obtained from the market is essential. Microbiological quality of some commercially packed and fresh fruit juice available in Dhaka city has been performed [5].

It is evident that no survey or assessment of guava fruit safety has yet been conducted in Bangladesh. Therefore, the objective of the present study was to isolate, identify and antimicrobial profile of bacteria isolated from fresh guava fruits sold at five markets in Mymensingh city.

## Materials and Methods

### Ethical approval

The present study was conducted during the period from July to December 2013, in the Bacteriology Laboratory of the Department of Microbiology and Hygiene, Faculty of Veterinary Science, Bangladesh Agricultural University (BAU), Mymensingh.

### Collection and transportation of samples

Guava samples were collected from KR market, Sheshmor, Kewatkhali, Meshubazar, and Nutunbazar (Table-1). Samples were collected in sterile polythene bags separately. The samples were transported carefully to the Bacteriology Laboratory of the Department of Microbiology and Hygiene, BAU, Mymensingh, for bacteriological analysis.

**Table-1:** Summary of samples used for the isolation of bacteria.

Name of markets	Sample number of guava collected	Total
KR market	5	25
Sheshmor	5	
Kewatkhali	5	
Meshubazar	5	
Nutunbazar	5	

### Processing of guava samples

Each guava sample was washed with 20 ml sterile PBS and transferred the washing sample into separate poly bag. A ten-fold serial dilution of the samples was performed in the nutrient broth.

### Determination of total viable count (TVC)

0.5 ml of each 10-fold diluted sample was transferred and spread on Plate Count Agar using a sterile pipette and a sterile glass spreader. Then, the plates were kept in an incubator at 37°C for 24-48 h. The number of colonies in a particular dilution was multiplied by the dilution factor to obtain TVC which was expressed as a mean log10 ± standard deviation colony-forming unit (cfu) per ml.

### Isolation of bacteria in pure culture

The isolation and identification of bacteria were performed according to the method described by Carter [6]. Samples were enriched in nutrient broth at 37°C for 24 h. The overnight cultures were streaked on SS agar (for *Salmonella* spp.), eosin methylene blue (EMB) (for *Escherichia coli*), BA (for *Bacillus* spp.), MS agar (for *Staphylococcus* spp.), and thiosulfate-citrate-bile salts-sucrose (TCBS) agar (for *Vibrio* spp.). The inoculated plates were incubated at 37°C for 24 h. A single colony was further subcultured until pure culture was obtained. Identification of bacteria was performed on the basis of colony morphology, Gram’s staining reaction, motility test, and biochemical tests.

### Molecular detection of E. coli by polymerase chain reaction (PCR)

#### DNA extraction

A pure bacterial colony of *E. coli* was mixed with 100 µl of distilled water which was boiled for 10 min and then immediately kept on ice for cold shock. Finally, centrifugation was done at 10,000 rpm for 10 min. The supernatant was collected and used as a DNA template for PCR.

#### Primers used for PCR

A genus-specific PCR was performed to amplify 16S rRNA of *E. coli* using previously published primers [7]. The list of primers is shown in Table-2.

**Table-2:** PCR primers with the sequence.

Primer	Sequence	Size (bp)
*E. coli* 16S (F)	5’-AATTGAAGAGTTTGATCATG3’	704
*E. coli* 16S (R)	5’-CTCTACGCATTTCACCGCTAC3’	

F=Forward, R=Reverse, bp=Base pair, PCR=Polymerase chain reaction, *E. coli*=*Escherichia coli*

### Antibiotic sensitivity test

Five isolates randomly selected from five genera were tested for antimicrobial drug susceptibility against five commonly used antibiotics by disc diffusion or Kirby-Bauer method [8]. Selection of 3–5 well-isolated colonies from the SS, BA, EMB, MS, and TCBS agar plate. Touched the top of colony with a loop and the growth is transferred into the nutrient broth. The broths were streaked onto Mueller-Hinton agar plates using sterile glass spreader homogeneously. Then, the antibiotic disc was placed onto Muller-Hinton agar and incubated at 37°C for 24 h. The plates were examined, and the diameter of the zones of inhibition was measured in mm from the edge of the disc to the edge of the zone using a meter ruler.

### Statistical analysis

The results of TVC of the bacteria of guava sold at local markets were analyzed for statistical significance using [9] multiple range test (SPSS 11.5, US). A p≤0.01 was considered to be statistically significant.

## Results

### TVC of guava

The bacterial load was the highest in Nutunbazar sample (log 6.62±0.02 cfu/ml), followed by Kewatkhali (log 6.60±0.03 cfu/ml), Meshubazar (log 6.58±0.04 cfu/ml), and KR market (log 6.57±0.03 cfu/ml). The lowest TVC was recorded in Sheshmor at BAU campus (log 6.56 cfu/ml). The bacterial load recorded in guava sold at all local markets did not vary and was found to be statistically significant (p=0.0007). The TVC of the guava samples collected from different markets is presented in Table-3.

**Table-3:** Total viable count (TVC) of guava sold at local market in Mymensingh

Markets name	TVC (mean log CFU±SD/ml)	p-value
Sheshmor	6.56±0.024	0.0007**
KR market	6.57±0.03	
Kewatkhali	6.60±0.03	
Nutunbazar	6.62±0.02	
Meshubazar	6.58±0.04	

** =Significant at 1% level of probability (P<0.01)

### Isolation of bacteria from guava surface samples

Five genera of bacteria such as *E. coli*, *Salmonella* spp., *Vibrio* spp., *Bacillus* spp., and *Staphylococcus* spp. were isolated from guava samples.

### Sugar fermentation and biochemical tests

The results of sugar fermentation tests using five basic sugars such as dextrose, maltose, lactose, sucrose, and mannitol are presented in Table-4. Acid and gas production was indicated by the change of color of phenol red from red to yellow and the presence of gas bubbles in Durham tube. The negative reaction was indicated by no change of color.

**Table-4:** Biochemical characteristics of *E. coli*, *Salmonella* spp., *Vibrio* spp. *Bacillus* spp., and *Staphylococcus* spp.

Sugar fermentation reaction profiles					MR test	VP test	Indole production test	Interpretation

DX	ML	L	S	MN				
AG	AG	AG	AG	AG	+	-	+	*E. coli*
A	A	-	-	A	+	-	-	*Salmonella* spp.
A	A	-	A	A	+	-	+	*Vibrio* spp.
AG	A	A	AG	AG	-	-	+	*Bacillus* spp.
A	A	A	A	A	+	+	-	*Staphylococcus* spp.

Legend: DX=Dextrose, ML=Maltose, L=Lactose, S=Sucrose, MN=Mannitol, A=Acid, AG=Acid and Gas, “+”=Positive, “”=Negative, “MR”=Methyl red, “VP”=Voges–Proskauer

### Distribution of bacterial isolates from guava sold at the local market

**Figure-1:**
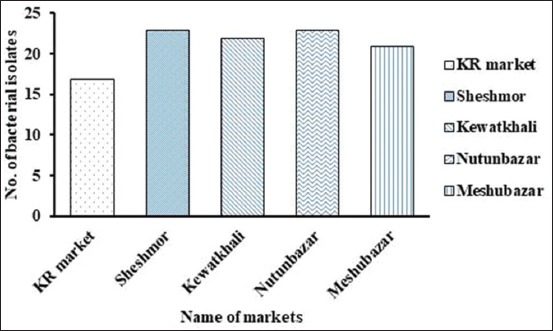
Number of bacterial isolates recovered from guava sold at local markets in Mymensingh. The highest number of bacteria were recovered from Sheshmor and Nutunbazar (23 isolates from 5 samples), followed by Kewatkhali (22 isolates from 5 samples), Meshubazar (21 isolates from 5 samples), and KR market (17 isolates from 5 samples).

The number of bacterial isolates detected in guavas sold at five local markets is presented in Figure-1.

Overall frequency distribution of *E. coli, Salmonella* spp., *Vibrio* spp., *Bacillus* spp., and *Staphylococcus* spp.

**Figure-2:**
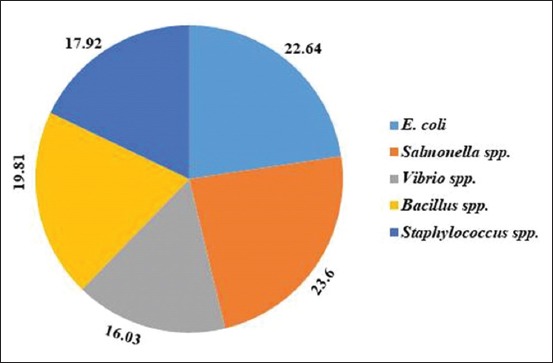
Overall percentage distribution of *Escherichia coli, Salmonella* spp., *Vibrio* spp., *Bacillus* spp., and *Staphylococcus* spp. in 25 guava samples sold at KR market, Sheshmor, Kewatkhali, Nutunbazar, and Meshubazar in Mymensingh.

A total of 106 bacterial isolates were obtained from 25 guava samples. The frequency distribution of *E. coli* was 22.64% (24 of 106), *Salmonella* spp. was 23.60% (25 of 106), *Vibrio* spp. was 16.03% (17 of 106), *Bacillus* spp. was 19.81% (21 of 106), and *Staphylococcus* spp. was 17.92% (19 of 106). Overall frequency distribution of *E. coli*, *Salmonella* spp., *Vibrio* spp., *Bacillus* spp., and *Staphylococcus* spp. is shown in a pie chart (Figure-2).

### Molecular detection of E. coli

**Figure-3:**
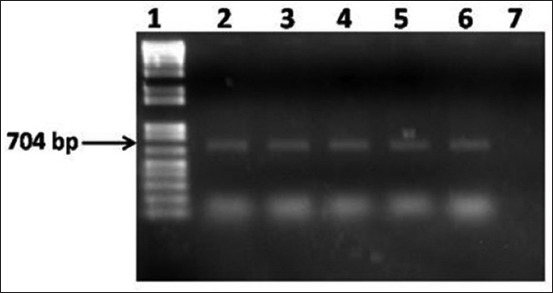
Polymerase chain reaction assay to amplify 16S rRNA of *Escherichia coli* isolates of guava sold at five local markets in Mymensingh. Lane 1: 100 bp-12 kb size DNA marker (TrackIt, Invitrogen, USA); lane 2: *E. coli* isolates of guava at KR market; lane 3: *E. coli* isolate of guava at Sheshmor; lane 4: *E. coli* isolate of guava at Kewatkhali; lane 5: *E. coli* isolate of guava at Nutunbazar; lane 6: *E. coli* isolate of guava at Meshubazar; lane 7: Negative control without DNA.

DNA extracted from five *E. coli* isolates was used in the PCR assay. PCR primers targeting 16S rRNA of *E. coli* amplified 704 bp fragments of DNA confirmed the identity of *E. coli* (Figure-3).

## Results of Antibiotic Sensitivity Tests

**Figure-4:**
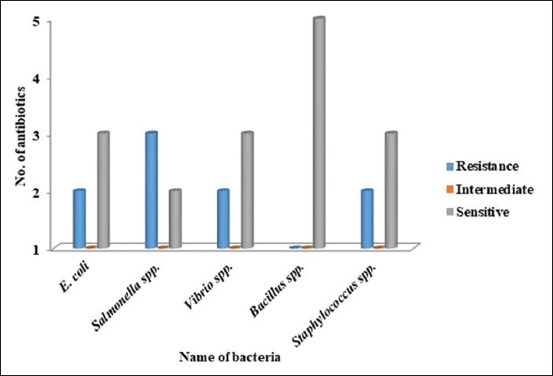
Summary of antibiogram profile of *Escherichia coli*, *Salmonella* spp., *Vibrio* spp., *Bacillus* spp., and *Staphylococcus* spp. against five antibiotics. *E. coli* was sensitive to three and resistant to two antibiotics, *Salmonella* spp. was sensitive to two and resistant to three antibiotics, *Vibrio* spp. was sensitive to three and resistant to two antibiotics, *Bacillus* spp. was sensitive to five but no resistant or intermediately resistant to any antibiotics, and *Staphylococcus* spp. was sensitive to three and resistant to two antibiotics.

A total of five isolates such as *E. coli*, *Salmonella* spp., *Vibrio* spp., *Bacillus* spp., and *Staphylococcus* spp. were subjected to antibiotic sensitivity assay. Summary of antibiogram profile of *E. coli*, *Salmonella* spp., *Bacillus* spp., *Vibrio* spp., and *Staphylococcus* spp. against antibiotics is presented in Figure-4.

## Discussion

Consumption of fresh fruit is increasing significantly in the recent days. Fruits usually do not receive any treatment, as a result, they are prone to get contaminated with foodborne and antibiotic-resistant bacteria that may cause public health hazards. In this study, TVC of bacteria of the washed sample of guavas sold at five local markets in Mymensingh ranged from log 6.56 to 6.62 cfu/ml. Oranusi *et al*. [10] stated that the acceptable microbial count in fruit juices suitable for human consumption ranged from log 4 cfu/ml to log 5 cfu/ml. The microbial load on guava recorded in this study exceeded the acceptable limit. In another study, total viable bacterial count in guava samples was observed within the range of 10^2^-10^7^ cfu/g [11].

In this study, 106 bacterial isolates belonged to five genera (such as *E. coli*, *Salmonella* spp., *Vibrio* spp., *Bacillus* spp., and *Staphylococcus* spp.) were identified. Eni *et al*. [12] isolated *Staphylococcus aureus, E. coli*, and *Salmonella* spp. from fruits and vegetables purchased from vendors. Mrityunjoy *et al*. [13] detected *Vibrio cholerae* in vegetables and fruits sold in Dhaka city. In this study, the distribution percentages of *E. coli*, *Salmonella* spp., *Vibrio* spp., *Bacillus* spp., and *Staphylococcus* spp. in guava were 22.64%, 23.60%, 16.03%, 19.81%, and 17.92%, respectively, which are nearly similar to the findings of Akhtar *et al*. [14], i.e. *Klebsiella pneumoniae* (25%), *E. coli* (21%), *Serratia marcescens* (12.5%), *Pseudomonas aeruginosa* (17%), *Bacillus cereus* (16.5%), and *S. aureus* (8%). Mukharjee *et al*. [15] reported 1.6% and 9.7% prevalence of *E. coli* in conventional and organic fruits grown by Minnesota farmers in the USA. Eni *et al*. [12] recorded 4.2%, 12.5%, and 29.2% prevalence of *E. coli, Salmonella* spp., and *S. aureus* in fruits and vegetables, respectively. Chikere and Azubuike [16] isolated Bacillus, *E. coli*, Enterobacter, *Salmonella*, Shigella, *Pseudomonas*, and *Staphylococcus* from guava samples in Nigeria. Contamination of guava with foodborne bacteria might be resulted from poor hygiene of the vendor, using microbial unsafe container, poor handling practice, and unsanitary market condition [17].

The occurrence of antibiotic-resistant bacteria in agricultural food staff is emerging throughout the world [17-19]. Antibiotic-resistant bacteria are known to spread from fruits and vegetables to human through the food chain. In this study, four genera of bacteria such as *E. coli*, *Salmonella* spp., *Vibrio* spp., and *Staphylococcus* spp. were found multidrug resistant (resistant against 2-3 antibiotics). Antibiotics against which bacterial isolates of guava were found resistant were ampicillin and cephalexin (*E. coli, Vibrio* spp., and *Staphylococcus* spp.), chloramphenicol, ampicillin, and cephalexin (*Salmonella* spp.). In the current study, no resistance was observed in the case of *Bacillus* spp. isolated from guava. Rashed *et al*. [19] identified multidrug-resistant *E. coli* and Staphylococci in fruit juice sample sold at Dhaka city. Nawas *et al*. [20] isolated multidrug-resistant *Salmonella* spp. and *Vibrio* spp. from salad vegetables. Antibiotic-resistant bacteria in plant food staffs are known to occur from the use of antibiotics in plant agriculture [21]. Application of animal manure to the agricultural field can also spread drug-resistant bacteria to plant [18].

## Conclusion

Data of this study suggest that guava sold at local markets of Mymensingh city harbors multidrug-resistant bacteria which underscore the need of the implementation of proper hygienic measures during pre- and post-harvest stages of production and at the time of transportation, storage, selling, and preparation of different products such as juices to safeguard public health.

## Authors’ Contributions

MMK and MAI designed the study. MARS and MMH collected and processed the samples for the isolation and identification of bacteria. MARS and MMH performed PCR and electrophoresis. MARS, MMH, and RAR did antibiotic sensitivity test. MARS, FAE, and MMK interpreted the results and analyzed the data. MARS, MAI, and MMK prepared the manuscript. All authors read and approved the final manuscript.
